# Clinical and genetic analyses of APMR4 syndrome caused by novel biallelic *LSS* variants

**DOI:** 10.3389/fnins.2024.1301865

**Published:** 2024-05-10

**Authors:** Qingyun Kang, Hui Kang, Jingwen Tang, Miao Wang, Haojiang Jiang, Zeshu Ning, Liwen Wu

**Affiliations:** ^1^Department of Neurology, The Affiliated Children’s Hospital of Xiangya School of Medicine, Central South University (Hunan children’s hospital), Changsha, China; ^2^Department of Orthopaedics, General Hospital of Central Theater Command, Wuhan, China

**Keywords:** *LSS*, APMR4, western blot, whole-exome sequencing, autosomal recessive diseases

## Abstract

Alopecia intellectual disability syndromes 4 (APMR4) caused by Lanosterol synthase (*LSS*) gene variants is a very rare autosomal recessive neuroectodermal syndrome. It is characterized by congenital alopecia and variable degrees of intellectual disability (ID), frequently associated with developmental delay (DD) and epilepsy. Currently, only three studies regarding *LSS*-related APMR4 have been reported, the pathogenesis of APMR4 is poorly understood. We studied one patient with *LSS*-related APMR4 who presented with severe intellectual disability, alopecia, early-onset epilepsy and developmental delay. She is absence of hair on the eyebrows, eyelashes, and scalp. Two novel *LSS* variants (c.401 T > G and c.369C > G) were detected with whole-exome sequencing (WES). Analysis via WB experiment indicated that c.369 > G reduced the protein expression level of *LSS*. Analysis of protein stability prediction showed a destabilizing for *LSS* caused by the variant c.401 T > G. This study is the first study in Asia to date. These findings expanded the variantal spectrum of *LSS*-related APMR4 and revealed the potential pathogenic mechanism of *LSS* gene variants.

## 1 Introduction

Alopecia-intellectual syndrome (APMR) is a group of neuro-dermal category autosomal recessive disorders, characterized by neurological deficits and ectodermal findings. Cardinal symptoms include loss of scalp hair, eyelashes, and eyebrows, as well as varying degrees (mild to severe) of intellectual disability (ID). Based on the degree of intellectual disability and associated phenotypic features, APMRs could be classified into four types. Clinical heterogeneity does exist among different APMR types. Patients with APMR1 and APMR2 have a mild to moderate degree of intellectual disability. Whereas, patients with APMR3 have severe intellectual disability. In patients with APMR4, the degree of intellectual disability was more extensive, ranging from mild to severe. Other symptoms, such as developmental delay and epilepsy, were observed in some patients with APMR1 and APMR4, but not with APMR2 or APMR3. AMPR is extremely rare, with a predicted estimated prevalence of less than 1:1,000,000 globally (Reza Sailani et al., 2017; Muzammal et al., 2021). Only a few families with APMR have been reported until now. APMR4 caused by Lanosterol synthase (*LSS*) gene variants is one type of the APMRs (Muzammal et al., 2021; Elaraby et al., 2022). It is first reported in 2019 and there are only nine families having been reported. The pathogenesis of APMR4 remains unknown. More cases with detailed clinical features and experimental data are needed. The gene *LSS* is of significant importance, with a mutation spectrum encompassing various types of mutations, such as missense mutations, nonsense mutations, and insertion–deletion mutations. These mutations result in aberrant function of *LSS*, thereby impacting the occurrence and progression of related diseases. Analysis of the mutation spectrum of *LSS* can enhance our understanding of its role in disease pathogenesis, providing more precise references for clinical diagnosis and treatment.

In this study, we report the clinical and molecular characteristics of one padiatric patient with alopecia, early-onset epilepsy, severe intellectual disability, developmental delay, and teeth dysplasia. Two compound heterozygous variants c.401 T > G (p.Val134Gly) and c.369\u00B0C > G (Try134*) of *LSS* were identified, which resulted in reduced expression of *LSS*, leading to the APMR4 phenotype in this patient. Literatures describing patients with *LSS*-related APMR4 were also collected and analyzed. We expanded the genotype spectrum of APMR4 and provided new insights into the potential pathogenic mechanism of this disease.

## 2 Materials and methods

### 2.1 Patients

This study was approved by the Ethics Committee of Hunan Children’s Hospital. A 1-year and 10-month-old female patient from the Department of Neurology of Hunan Children’s Hospital was recruited and included in the subsequent studies.

### 2.2 Genetic testing

Whole exome sequencing (WES) was performed on DNA samples from the patient and her parents. Leukocyte DNA was extracted from 3 mL of peripheral blood from each core family member. Libraries were constructed and sequenced using an Illumina HiSeq sequencer (Illumina Inc., San Diego, CA, United States). For suspected variants, population and literature databases were used for annotation, including dbSNP, 1,000 Genomes, ExAC, Clinvar., GnomAD, OMIM, and HGMD. Interpretation of variants was performed according to ACMG guidelines (Richards et al., 2015). Candidate variants identified by WES were confirmed by Sanger sequencing.

### 2.3 Conservative analysis

The human *LSS* protein and amino acid sequences of different species were retrieved from the Uniprot database, and the conservatism analysis was performed using the Align function provided by the database.

### 2.4 Quantitative polymerase chain reaction

Total RNA was extracted from the blood of the proband and two healthy controls by PAXgene total RNA extraction kit (FireGen, FG0411, China). First-strand cDNA was reversed by HiScript II 1st Strand cDNA Synthesis Kit (Vazyme, R212-02, China). Wild type and variant fragments were amplified with *LSS*-F (5’-ACATTGAGGATAAGTCCACCGT-3′) and *LSS*-R (5’-TCGTACCAGGTCAGGATCGTC-3′). GAPDH (internal control) was amplified with GAPDH-F (5’-CTGGGCTACACTGAGCACC-3′) and GAPDH-F (5’-AAGTGGTCGTTGAGGGCAATG-3′). Quantitative polymerase chain reaction (qPCR) was performed with AceQ qPCR SYBR Green Master Mix (Vazyme, Q111-02, China) according to the official guidelines. The relative expression of *LSS* (wild type and variant) was normalized to the expression of GAPDH.

### 2.5 Western blot

Proteins were extracted from blood samples collected from the proband and her parents using RIPA lysis and extraction buffer (P0013B, Beyotime, Shanghai, China). Protein concentrations were determined using a bicinchoninic acid (BCA) assay kit (P0010, Beyotime, Shanghai, China). Total protein (10 μg/lane) was loaded onto SDS-PAGE and transferred to a polyvinylidene difluoride membrane. After blocking with 10% skim milk for 1.5 h, the membrane was incubated with the primary antibody at 4°C overnight. The primary antibodies used in this study were polyclonal anti-*LSS* (13715-1-AP, proteintech, Illinois, United States) and monoclonal anti-GAPDH (AF0006, 1:1,000, Beyotime, Shanghai, China).

### 2.6 Protein stability prediction

Protein stability of wild type and mutant was predicted by the DynaMut,^1^ using the wild-type PDB file of *LSS* and the mutant loci (V134G). DynaMut is a tool for analyzing dynamic mutations in proteins. It utilizes molecular dynamics simulations to model the structure and dynamic changes of proteins, allowing for more accurate predictions of the effects of mutations on protein structure and function. By leveraging extensive experimental data and computational methods, DynaMut offers highly precise predictions of mutation effects, which can guide clinical treatment and drug design. In addition to predicting the impact of mutations on protein structure and function, DynaMut can also analyze the role of mutations in disease development, providing crucial information for disease diagnosis and treatment. Therefore, we have chosen DynaMut as our predictive tool.

### 2.7 Literature review

We searched the PubMed and Online Catalog of Human Genes and Genetic Disorders (OMIM) for previously reported *LSS*-related APMR4 cases using the *LSS* gene and APMR4 as keywords. The reported *LSS* variants and their corresponding clinical phenotypes were summarized. In terms of search criteria, we conducted literature searches using multiple databases including PubMed, EMBASE, and Web of Science. Key search terms included “APMR4 syndrome” and “*LSS* variants.” We specified a time frame for the literature search and selected articles relevant to our research topic. In the article selection process, we screened literature that met the criteria based on the research objectives and subjects. Articles that were not relevant to our research topic or did not meet the required methods were excluded to ensure the accuracy and reliability of the study. For quality assessment of the included studies, we conducted professional evaluations on each study, including assessment of study design, sample selection, and data analysis. We ensured the quality and credibility of the research, thereby enhancing the reliability and scientific validity of the study results.

## 3 Results

### 3.1 Case presentation

**Figure 1:**
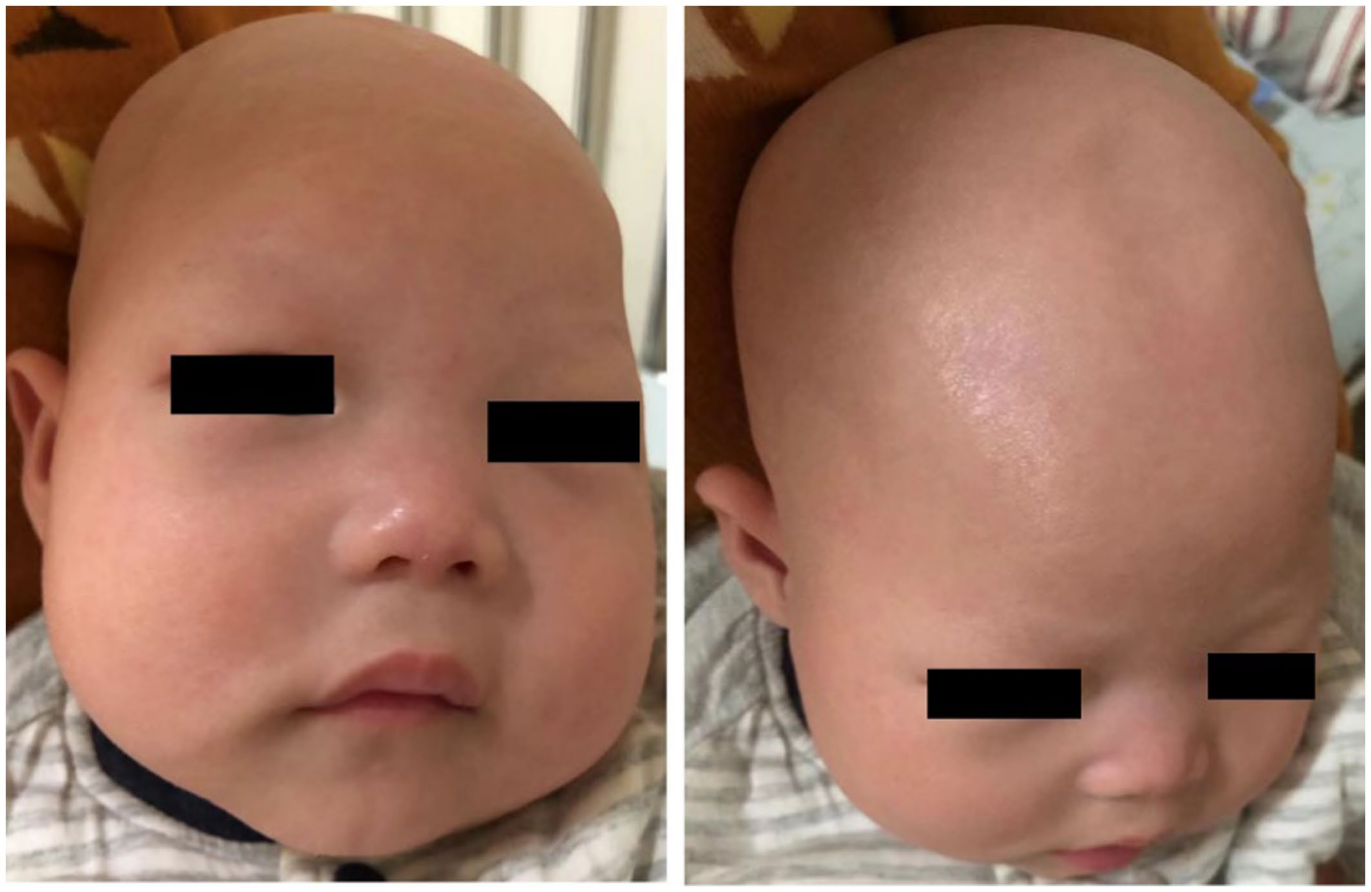
Clinical pictures of the patient with a phenotype of hypotrichosis.

**Figure 2:**
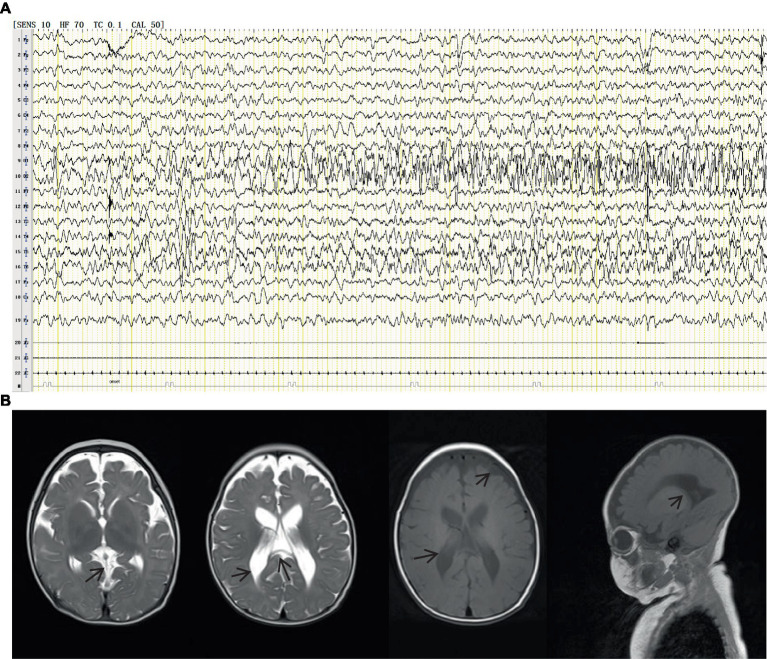
VEEG during the seizures showed that spike wave rhythms of medium to high amplitude first occurred at the electrodes in the occipital and posterior temporal regions, which later affected other regions **(A)**. Cranial magnetic resonance imaging (MRI) showed enlarged bilateral lateral ventricles and Vergae ventricle, the corpus callosum was thinned and the anterior temporal extracerebral space was widened bilaterally, with the bilateral lateral ventricles obviously deformed **(B)**.

A 1-year and 10-month-old Chinese girl was the first child of her healthy non-consanguineous parents. No other family history was apparently known except that her mother had a history of two spontaneous abortions. The patient was born at 40 weeks of gestation with normal Apgar scores, but she had a low birth weight of 2.2 kg. Hypotrichosis was noted at birth, including her eyelashes, hairs, and eyebrows (Figure 1). Moreover, her motor and cognitive developments were delayed from birth. At the age of 4 months, she was admitted to our department for unprovoked seizures. Physical examination on admission showed that she was insensitive to sound stimulation, had no eye contact, could not focus on or follow objects, and had hypotonia. Several facial abnormalities were observed, including a broad forehead, wide nasal base, and triangular face. After admission, she had multiple seizures, which were characterized by staring eyes, unresponsiveness to calls, spitting saliva from the mouth, and cyanosis of lips. The seizures lasted for several minutes and then relieved spontaneously. VEEG during the seizures showed that spike wave rhythms of medium to high amplitude first occurred at the electrodes in the occipital and posterior temporal regions, which later affected other regions (Figure 2A). Cranial magnetic resonance imaging (MRI) showed enlarged bilateral lateral ventricles and Vergae ventricle, the corpus callosum was thinned and the anterior temporal extracerebral space was widened bilaterally, with the bilateral lateral ventricles obviously deformed (Figure 2B). The Gesell Developmental Schedules was also tested. The development quotient (DQ) of adaptive behavior, fine motor, grand motor, personal social behavior and language were 50, 46, 22, 52, and 46, indicating Severe developmental delay. No other abnormalities were found. Therefore, the patient was diagnosed with epilepsy and hypoevolutism. Topiramate (TPM) was then titrated at an initial dose of 1 mg/kg/d and gradually increased to 6 mg/kg/d over one month. However, the patient still had recurrent seizures and had several epileptic states. She was treated with a combination of antiepileptic treatment with valproic acid (VPA) and TPM at her 6 months old. The starting dose of VPA was 10 mg/kg/d, with a gradually increased dose to 30 mg/kg/d for maintenance. The frequency of seizures decreased after the combination of VPA. But the patient still had seizures occasionally. At the age of 8 month, the patient was treated with clonazepam (starting dose 0.02 mg/kg/d, gradually increasing the dose to 0.2 mg/kg/d for maintenance) in combination with VPA and TPM. After clonazepam was increased to a titrated dose, the patient did not have seizures. TPM was tapered off at her 10 months old. And the patient did not have seizures during the titration period. The dose of clonazepam tablets was tried to be reduced when the patient was 11 months old. However, the patient had one recurrence of convulsive persistence during the clonazepam reduction period. The dose of clonazepam was gradually increased again to 0.2 mg/kg/d. The patient is currently treated with VPA and clonazepam and had no further seizures. Although the patient’s seizures were controlled, she still had significant developmental delay, up to now, she cannot stand alone, without meaningful communication. We observed that the phenotype of the patient was similar to that of patients with APMR4 syndrome previously reported, including low birth weight, developmental delay, and symptoms of epilepsy. However, through functional analysis of the newly discovered biallelic *LSS* variants, we found that these variants may have different impacts on function, providing a new perspective for our understanding of APMR4 syndrome.

### 3.2 Genetic analysis

**Figure 3:**
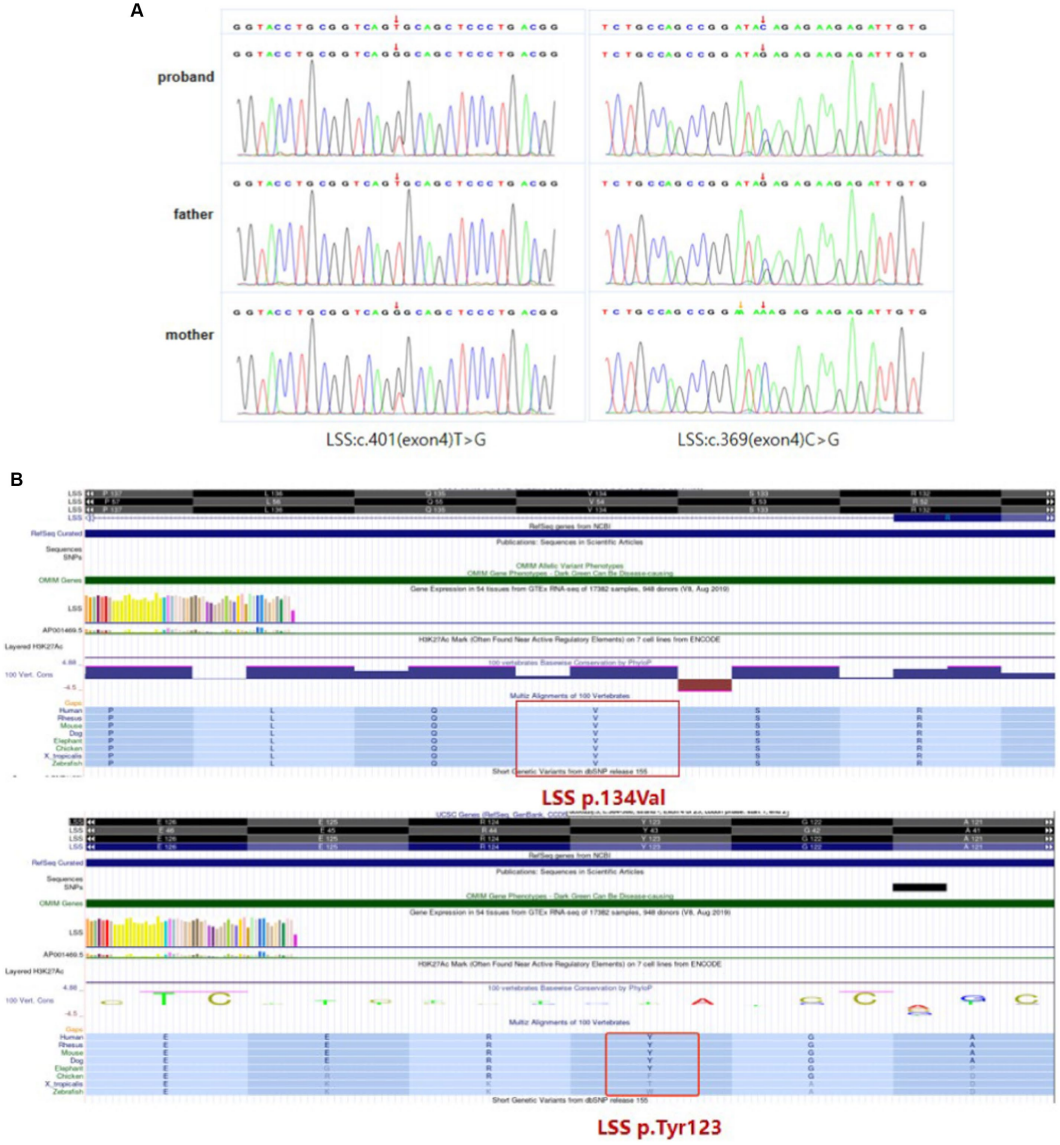
*LSS* mutation information. **(A)** Compound heterozygous variants c.401 T > G (p.Val134Gly) and c.369C > G (p.Tyr123*) of *LSS* gene identified in the patient. **(B)** The homology analysis shows that Val134 and Try123 of the *LSS* protein are conservative among different species.

WES was performed to identify causative variants. About 50.9 million clean reads were obtained with an average sequencing depth of 112.43X and an average coverage of 97.60% for target regions larger than 30X. Finally, two novel compound heterozygous variants c.401 T > G (p.Val134Gly) and c.369C > G (p.Tyr123*) of *LSS* gene were identified, which was inherited from her mother and father, respectively (Figure 3A). The paternal variant c.369C > G in exon 4 introduced a premature terminator (p.Try134*), which resulted in a truncating protein without normal function (PVS1). It was absent from controls (1,000 Genomes, ExAC, gnomAD, and CNGB) (PM2). So we could classify it as a likely pathogenic variant according to the ACMG guidelines. The maternal missense variant c.401 T > G in the same exon led to an amino acid change (p.Val134Gly). It located in disease-causing hotspots (PM1) and was not present in the ExAC, dbSNP, or 1,000 Genomes databases (PM2). It was in *trans* with the likely pathogenic variant c.369C > G (PM3) and was predicted to be a deleterious variant by several computational software programs (PP3). According to the ACMG guidelines, the variant c.401 T > G was also defined as a likely pathogenic variant (PM1 + PM2 + PM3 + PP3). Sanger sequencing confirmed the existence of each mutation. The homology analysis showed that the *LSS* proteins Val134 was conservative among different species (Figure 3B).

### 3.3 Validation of the pathogenic variants

**Figure 4:**
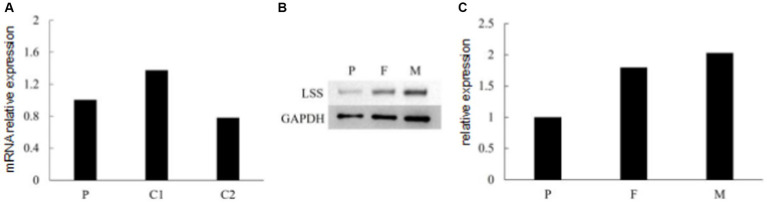
**(A)** RNA expression of *LSS* detected by qPCR. **(B,C)** Immunoblot of *LSS* proteins expressed in the patient and her parents.

**Figure 5:**
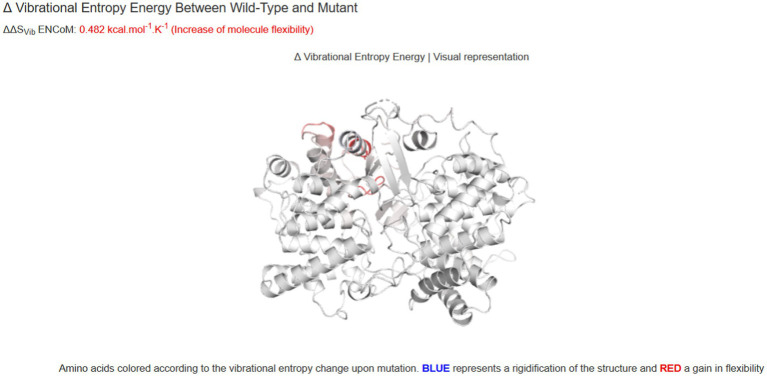
The prediction of the thermodynamic stability of the proteins using the DynaMut database and multi-analysis data tools suggests that *LSS* variant c.401 T > G (p.Val134Gly) caused protein destabilization and improved molecular flexibility in the corresponding conformational space.

To investigate the effect of two variants identified in the patient, we performed qPCR and western blot. No significant changes were observed at the RNA level (Figure 4A), suggesting that neither variant affects the transcription of *LSS*. A significant down-regulation of *LSS* expression (~50%) was observed in western blot (Figures 4B,C), indicating that the nonsense variant might affect the protein of *LSS*. To further investigate the effect of the missense variant of *LSS* [NM_002340.6: c.401 T > G, (p.Val134Gly)], the stability of the variant *LSS* was evaluated. The results showed a decrease in the stability of the *LSS* caused by this variant (DynaMut: −1.349 kcal/mol), and the other four prediction tools showed a similar downward trend (ENCoM: −0.386 kcal/mol; mCSM: −2.042 kcal/mol; SDM: −2.370 kcal/mol; DUET: −2.392 kcal/mol) (Figure 5). Based on these results, we can conclude that these mutations may affect the function of the *LSS* protein by influencing its stability. However, further functional analysis is still required to comprehensively understand the mechanisms by which these mutations impact protein function.

### 3.4 Summary of reported *LSS*-related APMR4 cases

The OMIM and PubMed databases were searched for literatures related to “*LSS*” and “APMR4” included up to March 2023. A total of 3 publications involving 16 patients with *LSS*-related APMR4 were reported. Fourteen *LSS* variants were identified in these 16 patients, which included 9 missense variants, 2 nonsense variants, 2 splicing variants and 1 frameshift variant. All 16 patients had congenital alopecia varying degrees of developmental delay, among which 7 patients had epilepsy, 2 patients had ectodermal phenotypes, and some patients had MRI abnormalities. Detailed clinical features of the reported patients were shown in Table 1.

**Table 1:** Clinical features of individuals with *LSS*-related APMR4.

Family	Case	Sex	Age	Nucleotide variant	Amino acid variant	Congenital alopecia	Intellectual disability	Intellectual disability degree	Congenital cataracts	Epilepsy	Ectodermal phenotypes	Total cholesterol level	Brain MRI anomalies	Antiepileptic drugs	Seizure control	Reference
F1	1	F	1 year	c.625A > T;c.423G > A	p.Asn209Tyr p.Trp141*	√	√	ND	×	ND	×	Normal	ND	ND	ND	Romano et al. (2018)
	2	M	ND			√	√	ND	×	ND	×	Normal	ND	ND	ND	Romano et al. (2018)
F2	3	F	7 years	c.1547A > G;c.2114C > A	p.Asn516Ser p.Thr705Lys	√	√	Severe	×	√	×	Normal	√	ND	Controlled	Besnard et al. (2019)
	4	F	3 years			√	√	Severe	×	√	×	Normal	√	Zonisamide	Controlled	Besnard et al. (2019)
F3	5	M	19 years	c.779G > C;c.1194 + 5G > A	p.Arg260Pro Splice p.?	√	√	Moderate	×	ND	√	ND	ND	ND	ND	Besnard et al. (2019)
	6	F	10 years			√	√	Moderate	×	ND	√	ND	ND	ND	ND	Besnard et al. (2019)
F4	7	F	12 years	c.1109 + 2 T > C	Splice p.?	√	√	Mild	×	ND	×	Low	ND	ND	ND	Besnard et al. (2019)
	8	M	10 years			√	√	Severe	×	√	×	Normal	√	ND	ND	Besnard et al. (2019)
F5	9	M	2 years	c.857A > G;c.1810C > T	p.Tyr286Cys p.Arg604*	√	√	Severe	×	√	√	ND	ND	Levetiracetam	ND	Besnard et al. (2019)
	10	M	6 months			√	√	Severe	×	√	√	ND	ND	ND	ND	Besnard et al. (2019)
F6	11	M	14 years	c.1417dup;c.41A > G	p.His473Profs*32 p.Tyr14Cys	√	√	Severe	×	√	×	Normal	√	Pyridoxine	Controlled	Besnard et al. (2019)
F7	12	M	4 years	c.35G > A	p.Gly12Asp	√	√	Moderate	×	√	×	Normal	ND	Levetiracetam + valproic acid	Controlled	Besnard et al. (2019)
F8	13	F	11 years	c.1955C > T; unidentified but allelic imbalance	p.Thr652Ile	√	√	Severe	×	√	×	ND	√	ND	ND	Besnard et al. (2019)
F9	14	F	2 months	c.1609G > T	p.Val537Leu	√	√	ND	×	ND	×	Normal	ND	ND	ND	Elaraby et al. (2022)
	15	F	2 years and 6 months			√	√	ND	×	ND	×	Normal	√	ND	ND	Elaraby et al. (2022)
	16	ND	Fetus			√	ND	ND	×	ND	×	Normal	ND	ND	ND	Elaraby et al. (2022)
F10	17	F	1 year and 10 months	c.401 T > G;c.369\u00B0C > G	p.Val134Gly p.Try134Ter	√	√	Severe	×	√	√	Normal	√	Valproic acid, topiramate, clonazepam	Controlled	This study

F, female; M, male; ND, no description.

## 4 Discussion

Cholesterol metabolic pathways are of fundamental importance to the metabolism in the human body. Their intermediate and end metabolites are important for the structure and function of the central nervous system and hair follicle morphology (Ruf et al., 2004; Mitsche et al., 2015). Variants in any enzymes involved in the pathways could cause insult to the pathways and induce disorders of the body’s functions. Lanosterol synthase encoded by the *LSS* gene is a key enzyme in the cholesterol synthesis pathway (Huff and Telford, 2005), and lanosterol is the first sterol intermediate of the pathway (Blanc et al., 2011). In neurology, the function of *LSS* primarily involves cholesterol synthesis and the formation of neuronal cell membranes. Variations in *LSS* can disrupt the cholesterol synthesis pathway, thereby affecting the normal function of neuronal cells. *LSS* mutations can lead to abnormalities in the cholesterol synthesis pathway, subsequently impacting the membrane structure and function of neuronal cells, resulting in neurological symptoms such as hair loss. Additionally, certain *LSS* mutations may directly affect the function of neuronal cells, leading to neurological disorders such as intellectual disabilities.

Since Zhao et al. (2015) reported two families of congenital cataracts caused by *LSS* variants, which is the earliest phenotype of *LSS* gene mutation, *LSS* gene variation was detected in several families with hypotrichosis. Romano et al. (2018) reported three families with hypotrichosis simplex related to *LSS* variants and described intellectual disability in two members of one family from Switzerland. The authors considered the intellectual disability a coincidence at that time. In the next year, Besnard et al. (2019) reported 11 patients with both hypotrichosis and intellectual disability from 7 families. Through stability prediction of the case variant and comparison analysis with mutations previously reported in the literature, we found that the *LSS* protein stability decreased due to the case variant (DynaMut: −1.349 kcal/mol), showing some differences compared to other mutations reported earlier. This discovery provides important clues for further understanding the impact of this novel biallelic *LSS* variant on the pathogenesis of the disease. In the case presentation section, detailed descriptions of the clinical manifestations and treatment process of a 1-year-10-month-old Chinese girl are provided. The patient exhibited symptoms such as low birth weight and sparse hair at birth, and various abnormal manifestations during development, including seizures, cognitive and motor developmental delays. After a series of examinations and treatments during hospitalization, the patient’s seizures were partially controlled, but severe developmental delay still persisted. Therefore, the phenomenological spectrum of *LSS* variation was expanded to APMR4 syndrome.

Consistent with the two reports, the clinical phenotypes of our patients include alopecia and severe intellectual disability as well as early-onset epilepsy. Our patient has two compound heterozygous variants, c.401 T > G (p.Val134Gly) and c.369C > G (p.Tyr123*), of the *LSS* gene, which has not yet been reported. Our Western blot analysis assays indicated that the nonsense variant c.369C > G reduced the protein expression of the *LSS* gene. The prediction of the thermodynamic stability of the proteins using the DynaMut database and multi-analysis data tools suggests that *LSS* variant c.401 T > G (p.Val134Gly) caused protein destabilization and improved molecular flexibility in the corresponding conformational space. We have identified a certain correlation between different types of mutations in the *LSS* gene and the clinical manifestations in patients. Specifically, we have observed that some novel biallelic *LSS* mutations are associated with the onset of APMR4 syndrome, further supporting the link between the *LSS* gene and this syndrome. Furthermore, we have found a wide spectrum of heterogeneity in the phenotypic manifestations of *LSS* mutations, with patients exhibiting diverse clinical features including, but not limited to, abnormalities in the nervous system, immune system, and metabolic system. These findings provide important clues for further investigating the relationship between *LSS* mutations and APMR4 syndrome. Collectively, clinical examination and genetic analysis of our patient confirmed the diagnosis of APMR4 syndrome.

Biallelic variants in the *LSS* gene cause a broad spectrum of disease phenotypes. Phenotypes such as congenital cataract, hypotrichosis, and APMR4 syndrome are commonly reported; microcephaly, ichthyosis, acanthosis, cleft palate, hypospadias, sparse teeth, micropenis, linguistic disturbance, learning disability, hearing impairment, impaired concentration, and growth retardation are also observed in some patients (Chen and Liu, 2017; Wada et al., 2020; Cesarato et al., 2021; Hua et al., 2021; Elaraby et al., 2022). The phenotypes of *LSS* variation vary from severe neurological syndrome to a mild condition with hypotrichosis simplex only. Different *LSS* variants among families may explain the phenotypic heterogeneity, while different individuals in one family may have different clinical phenotypes. Epigenetic modification and other modifier genes may contribute to phenotypic heterogeneity, but the exact mechanism underlying clinical heterogeneity is still unclear (Cesarato et al., 2021; Elaraby et al., 2022). Further experimental studies are required to investigate the pathogenesis and mechanism of the phenotypic variation in *LSS* deficiency.

Romano et al. (2018) noted that variant in the C terminal domain was associated with ocular manifestations while variants in the N terminal domain were associated with hair loss. However, Elaraby et al. (2022) reported no ocular manifestations in the patient with a variant in the C terminal domain. According to the current *LSS* gene variation-related disease spectrum, it is still impossible to infer the clinical phenotype from the genotype. The correlation between genotype and phenotype is not clear. This may be attributable to the lack of sufficient genotype and phenotype data.

Among the previously reported patients with APMR4 syndrome, dysplasia of the corpus callosum is the most common abnormality in head MRI and is also found in our case. Eight of the 11 patients with AMPR4 reported by Besnard et al. (2019) had epilepsy, and most of them experienced seizures in the neonatal period; most of the cases were intractable epilepsy. In our case, the onset of epilepsy was relatively late, frequent seizures began at the age of four months, and status epilepticus occurred many times during the disease course. The epilepsy of our patient was well controlled after trying various antiepileptic drugs among which clonazepam showed a good therapeutic effect. In this study, we observed that patients with *LSS*-related APMR4 syndrome adopted a novel drug treatment regimen, successfully controlling epileptic seizures. Compared to previously reported treatment regimens, the drug regimen used in this study demonstrated superior efficacy, effectively managing the patients’ epileptic symptoms. This indicates that the drug has a significant effect in epileptic patients, playing a crucial role in improving patients’ quality of life and disease management. The drug may exert its anti-epileptic effects through various pathways such as modulating neuronal excitability, influencing neurotransmitter release, or regulating synaptic transmission between neurons. Additionally, the drug may also reduce seizure frequency and severity by alleviating neuronal hyperexcitability, inhibiting abnormal electrical activity, and other mechanisms.

In conclusion, this study first describes APMR4 syndrome in the Chinese population, summarizes the ten molecularly proven families reported to date, and expands the clinical and variant spectrum of the syndrome. Our results emphasize the importance of genetic testing of patients with APMR4 syndrome, to provide prenatal diagnosis and proper genetic counseling for families with the syndrome.

## Data availability statement

The original contributions presented in the study are included in the article/Supplementary material, further inquiries can be directed to the corresponding authors.

## Ethics statement

The studies involving humans were approved by the Ethics Committee of Hunan Children’s Hospital. The studies were conducted in accordance with the local legislation and institutional requirements. Written informed consent for participation in this study was provided by the participants’ legal guardians/next of kin. Written informed consent was obtained from the individual(s), and minor(s)’ legal guardian/next of kin, for the publication of any potentially identifiable images or data included in this article.

## Author contributions

QK: Writing – original draft. JT: Data curation, Writing – review & editing. MW: Data curation, Writing – review & editing. HJ: Writing – review & editing. ZN: Methodology, Supervision, Writing – review & editing. LW: Methodology, Supervision, Writing – review & editing. HK: Methodology, Writing – review & editing.

## Footnotes

^1^https://biosig.lab.uq.edu.au/dynamut/prediction
