# Supplementary figures and images for: Clinical and genetic analyses of APMR4 syndrome caused by novel biallelic LSS variants
Source: Front Neurosci. 2024 May 10;18:1301865. doi: 10.3389/fnins.2024.1301865 (PMC11116803; doi:10.3389/fnins.2024.1301865)

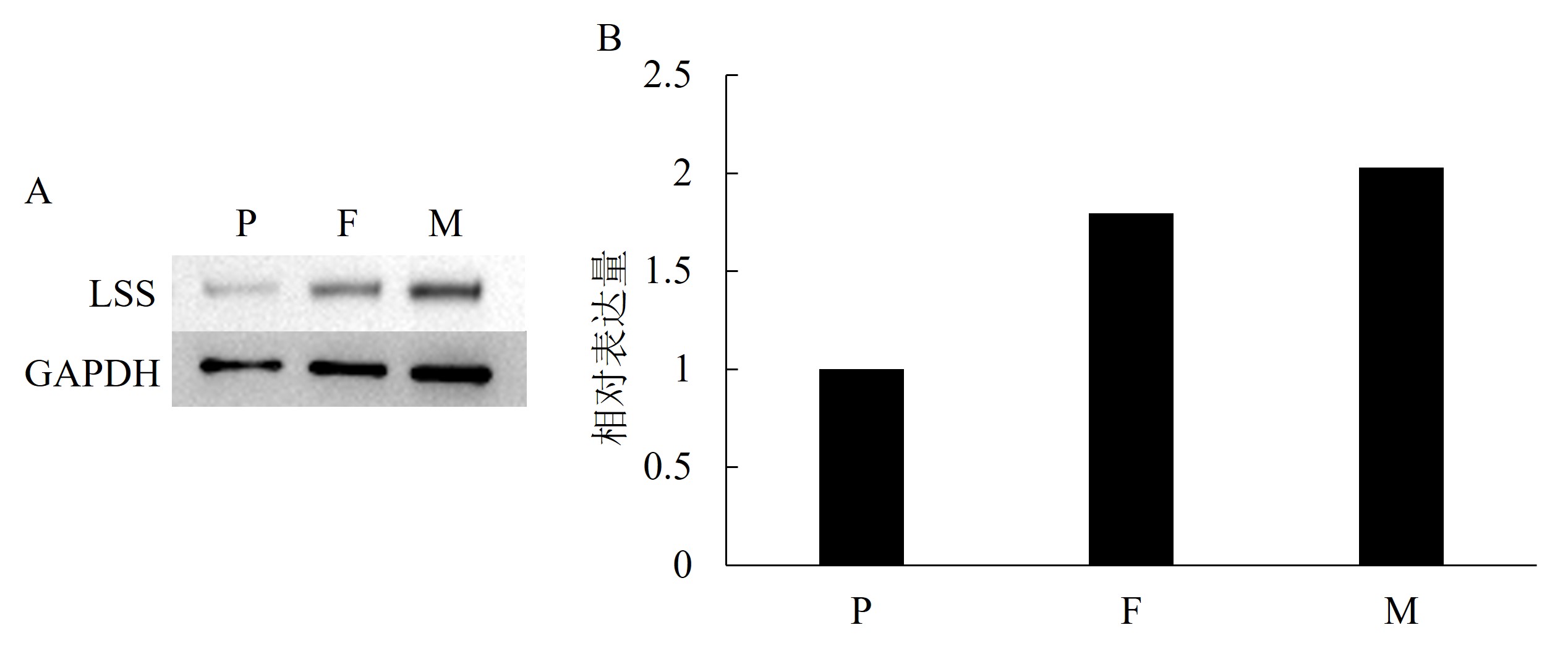

Supplement: Supplementary file 1 [file Data_Sheet_1.ZIP › 【Raw data】LSS_PBMC-WB+qPCR/figures -for report/LSS- Immunoblot of LSS proteins expressed in the patient and her parents..jpg]

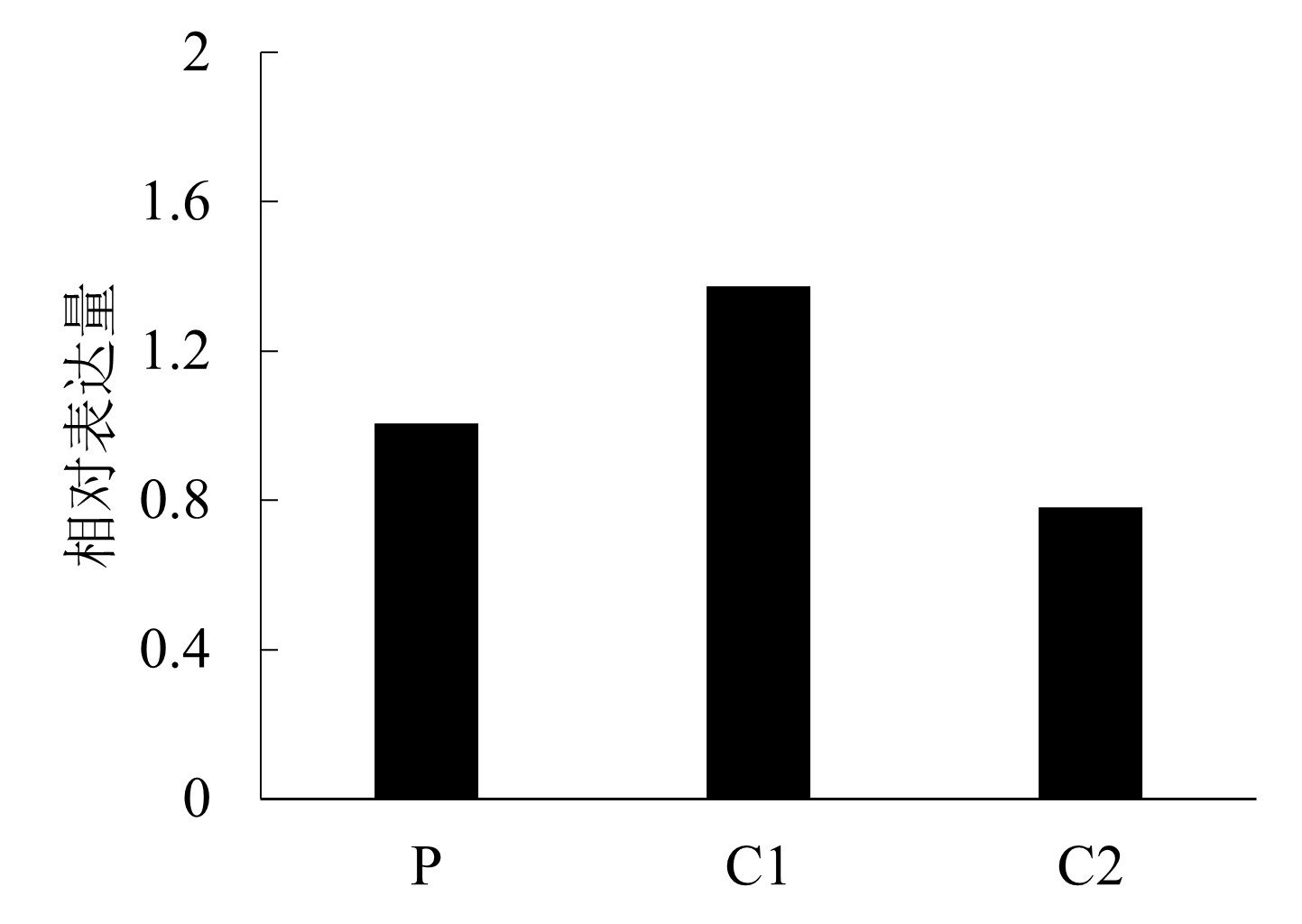

Supplement: Supplementary file 1 [file Data_Sheet_1.ZIP › 【Raw data】LSS_PBMC-WB+qPCR/figures -for report/LSS-RNA expression of LSS detected by qPCR.jpg]

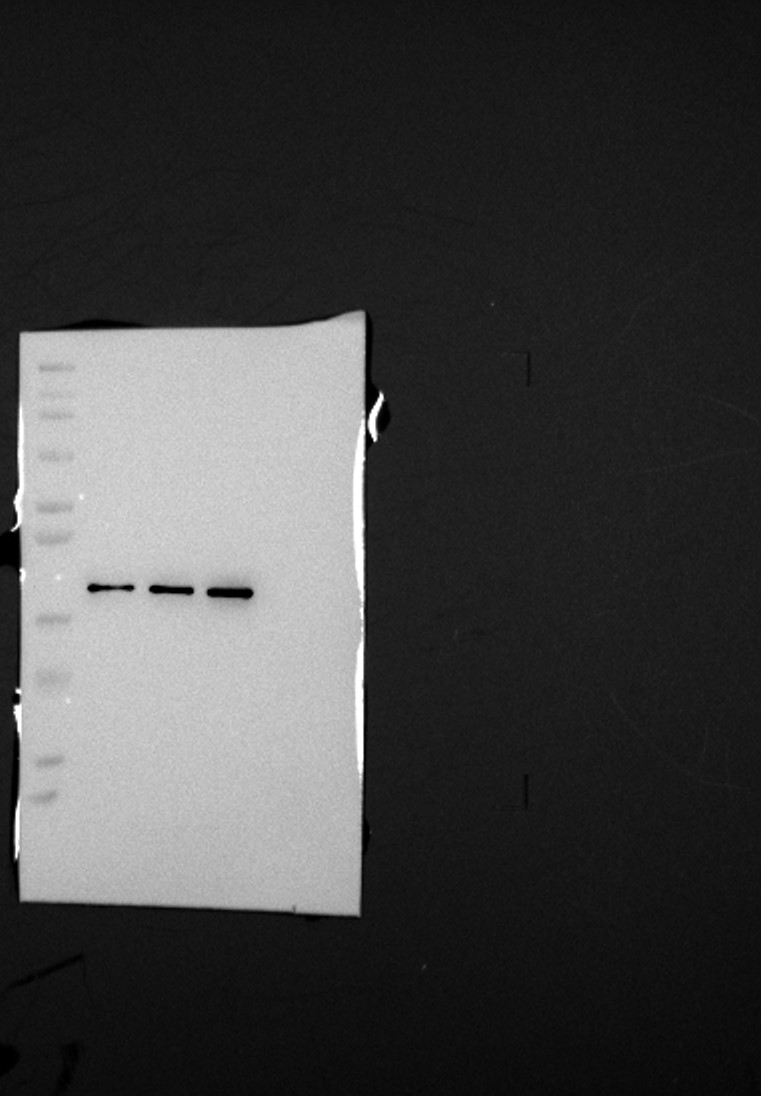

Supplement: Supplementary file 1 [file Data_Sheet_1.ZIP › 【Raw data】LSS_PBMC-WB+qPCR/WB -raw figures/GAPDH.jpg]

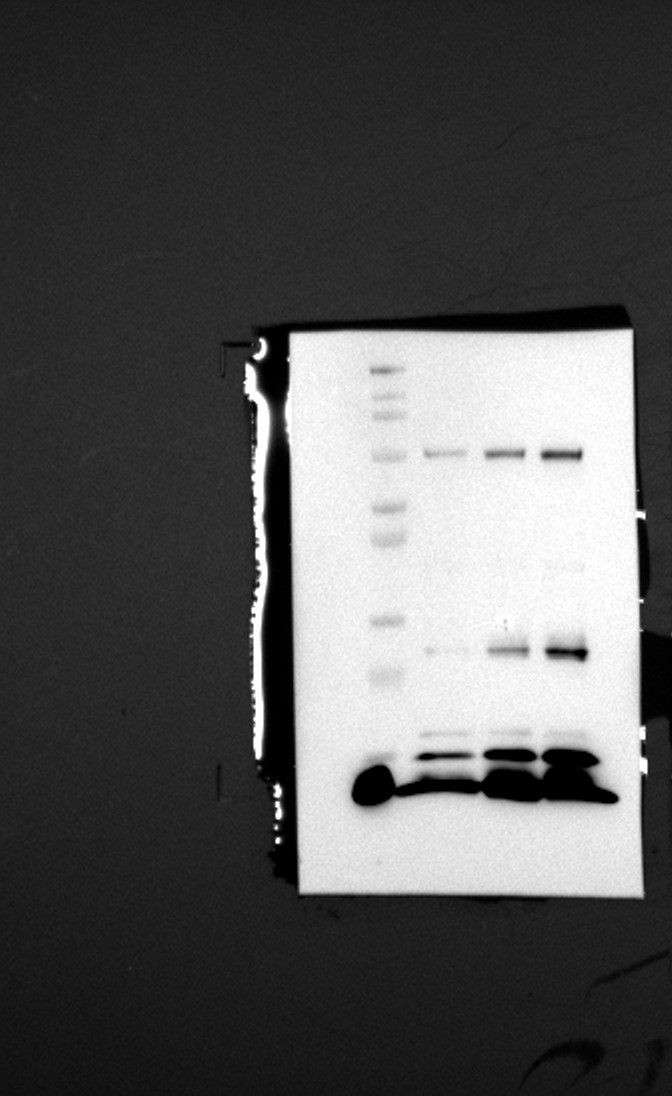

Supplement: Supplementary file 1 [file Data_Sheet_1.ZIP › 【Raw data】LSS_PBMC-WB+qPCR/WB -raw figures/LSS.jpg]
